# Autophagy Promotes Cigarette Smoke-Initiated and Elastin-Driven Bronchitis-Like Airway Inflammation in Mice

**DOI:** 10.3389/fimmu.2021.594330

**Published:** 2021-03-22

**Authors:** Hua-Qiong Huang, Na Li, Dan-Yang Li, Du Jing, Zheng-Yuan Liu, Xu-Chen Xu, Hai-Pin Chen, Ling-Ling Dong, Min Zhang, Song-Min Ying, Wen Li, Hua-Hao Shen, Zhou-Yang Li, Zhi-Hua Chen

**Affiliations:** ^1^ Key Laboratory of Respiratory Disease of Zhejiang Province, Department of Respiratory and Critical Care Medicine, Second Affiliated Hospital of Zhejiang University School of Medicine, Hangzhou, China; ^2^ State Key Laboratory of Respiratory Disease, Guangzhou Medical University, Guangzhou, China

**Keywords:** autophagy, chronic obstructive pulmonary disease-COPD, MMP12, inflammation, elastin

## Abstract

Cigarette smoke (CS)-induced macrophage activation and airway epithelial injury are both critical for the development of chronic obstructive pulmonary disease (COPD), while the eventual functions of autophagy in these processes remain controversial. We have recently developed a novel COPD mouse model which is based on the autoimmune response sensitized by CS and facilitated by elastin. In the current study, we therefore utilized this model to investigate the roles of autophagy in different stages of the development of bronchitis-like airway inflammation. Autophagic markers were increased in airway epithelium and lung tissues, and *Becn^+/-^* or *Lc3b^-/^* ^-^ mice exhibited reduced neutrophilic airway inflammation and mucus hyperproduction in this COPD mouse model. Moreover, treatment of an autophagic inhibitor 3-methyladenine (3-MA) either during CS-initiated sensitization or during elastin provocation significantly inhibited the bronchitis-like phenotypes in mice. Short CS exposure rapidly induced expression of matrix metallopeptidase 12 (MMP12) in alveolar macrophages, and treatment of doxycycline, a pan metalloproteinase inhibitor, during CS exposure effectively attenuated the ensuing elastin-induced airway inflammation in mice. CS extract triggered MMP12 expression in cultured macrophages, which was attenuated by autophagy impairment (*Becn^+/-^* or *Lc3b^-/^* ^-^) or inhibition (3-MA or Spautin-1). These data, taken together, demonstrate that autophagy mediates both the CS-initiated MMP12 activation in macrophages and subsequent airway epithelial injury, eventually contributing to development COPD-like airway inflammation. This study reemphasizes that inhibition of autophagy as a novel therapeutic strategy for CS-induced COPD.

## Introduction

Chronic obstructive pulmonary disease (COPD) is currently the fifth public health burden and the third leading cause of death (1, 2). This disease is characterized by persistent respiratory symptoms and partly reversible airflow obstruction, which encompasses two major clinical phenotypes, chronic bronchitis and emphysema (3). Cigarette smoking is the major risk factor of COPD, eventually causing chronic airway inflammation, mucus hyperproduction, destruction of lung tissue, and small airway remodeling (4, 5). However, the cellular and molecular mechanisms mediating cigarette smoke (CS)-induced COPD pathogenesis remain largely unknown.

Autophagy is a dynamic process responsible for the turnover of cellular organelles and long-lived proteins, thereby playing an essential role in cellular homeostasis and adaptation to adverse environments (6). Contrarily, autophagy could mediate cellular damage, contributing to disease development (7–9). We and others have demonstrated that autophagy mediates the airway epithelial cell death, mucus hyperproduction, and inflammatory responses in the context of CS-induced COPD (10–15), as well as in other airway disorders induced by environmental particulate matter or allergic cytokines (16–18). However, it remains controversial that CS may impair autophagy in airway epithelium, leading to cellular senescence, lung aging, and emphysema development (19–21). Monick et al. have demonstrated that CS impaired autophagy in alveolar macrophages of COPD patients, and this autophagy impairment resulted in decreased protein aggregate clearance, dysfunctional mitochondria, and defective delivery of bacteria to lysosomes (22). However, the exact functions of autophagy in pulmonary macrophages in CS-induced COPD pathogenesis remain largely unknown.

We have recently developed a new COPD mouse model based on the CS-initiated and elastin-driven autoimmunity (23). In this model, CS exposure rapidly induces expression of macrophage metalloproteinase (MMP12), which degrades endogenous elastin to active peptides, thereby triggering the autoimmune response. Subsequent elastin provocation induces apparent bronchitis-like phenotypes in the mouse lungs, including neutrophilic airway inflammation, Th17 response, and mucus hyperproduction (23). This model thus provides ideal approaches to study the molecular mechanisms in different stages of COPD inflammation, i.e., early CS-initiated alveolar macrophage activation and late airway epithelial injury.

This study aims to explore the eventual functions of autophagy in the overall airway inflammation induced by CS exposure following elastin challenge. We demonstrate that autophagy promoted the bronchitis-like airway inflammation, likely through facilitation of both early MMP12 induction in macrophages and late airway epithelial injury.

## Materials And Methods

### Chemicals and Reagents

Antibodies against ACTB (1:1000 dilution, sc-130300, mouse monoclonal, Santa Cruz Biotechnology, Dallas, TX, USA), LC3B (1:1000 dilution, L7543, rabbit polyclonal, Sigma-Aldrich, St. Louis, MO, USA), SQSTM1 (1:1000 dilution, 4814, rabbit monoclonal, Cell Signaling Technology, Danvers, MA, USA), MMP12 (1:2000 dilution, ab52897, rabbit monoclonal, Abcam, Cambridge, UK) were used. Spautin-1 (S7888) and 3-MA (S2767) were from Selleck (Houston, TX, USA). Doxycycline was purchased from Sigma-Aldrich (1226003, St. Louis, MO, USA). Mouse Elastin Peptide were from Elastin Products Company (CB573, Owensville, MO, USA).

### Cell Culture

THP-1 cells were purchased from American Type Culture Collection (TIB-202) and were maintained in RPMI 1640 (R8758, Sigma-Aldrich, St. Louis, MO, USA) with 10% FBS (v/v) (A3160801, Thermo Fisher Scientific, Waltham, MA, USA), 50 U/ml penicillin, and 50 U/ml streptomycin (C0222, Beyotime, Shanghai, China).

### Cell Viability Assay

The cytotoxic effects of CSE and chemical inhitors on THP-1 and BMDMs were determined by an assessment of cell viability using monosodium salt (WST-8) with a Cell Counting Kit-8 (CCK-8) assay (Cat. C0037, Beyotime, Shanghai, China). THP-1 cells or BMDMs cultured in 96-well plates were exposed to different concentrations of CSE for 24h or 1% CSE for different time with or without chemical inhibitors as indicated. The control-cultured cells were incubated with culture medium for 24 h. 90 μl of cell suspension was incubated with 10 μl of WST solution for 2 h at 37 °C in a 5% CO_2_ atmosphere, and then the assay was stopped. The absorbance of the samples at a wavelength of 450 nm was measured using a microplate reader (MULTISKAN MK3, Thermo Fisher Scientific, Waltham, MA, USA).

### Preparation of Bone Marrow-Derived Macrophages

The bone marrow-derived macrophages were prepared as described previously (24) with some minor adaptions. Briefly, 6–8 weeks old mice were sacrificed by cervical dislocation and soaked in 75% ethanol. The femurs and tibias were harvested and the bone marrow cells from all bones were flushed out. After centrifuging for 5 min at 400g, erythrocytes were eliminated using RBC Lysis Buffer (555899, BD Biosciences, San Jose, CA, USA). The remaining cells were seeded in plates and were incubated in RPMI 1640 containing 10% (v/v) heat-inactivated FBS and 20 ng/ml recombinant mouse M-CSF (CB34, Novoprotein Scientific Inc., Shanghai, China) for 5 days to form proliferative non-activated cells.

### Animals

C57BL/6JSlac male mice aged 6-8 weeks were purchased from the Shanghai Laboratory Animal Center (Shanghai, China). *Becn1^+/-^* mice on the background of 129X1/SvJ and backcrossed to C57BL/6 for 50 generations were kindly provided by Dr. Beth Levine, University of Texas Southwestern Medical Center. Heterozygous mice were bred to C57BL/6JSlac wildtype mice, offspring with a brown coat were considered heterozygous for their unique phenotype. *Lc3b^-/-^* mice on the mixed B6;129P2 genetic background were purchased from Jackson Laboratory (Stock No: 009336, Bar Harbor, ME, USA). Heterozygous mice were bred together to obtain the homozygous ones. Genotyping primers for *Lc3b^-/-^* mice (Wild type Forward 5’-3’: GAC ACC TGT ACA CTC TGA TGC ACT; Common 5’-3’: CCT GCC GTC TGC TCT AAG CTG; Mutant Forward 5’-3’: CCA CTC CCA CTG TCC TTT CCT AAT) were synthesized by Sangon Biotech, Shanghai, China. All mice were maintained and bred in the animal facility of the laboratory animal center of Zhejiang University. Male mice aged 6-8 weeks were used for each experiment. All experimental biosecurity procedures were adhered to the rules and regulations of laboratory animal center of Zhejiang University and Key Laboratory of Respiratory Disease of Zhejiang Province. All experimental protocols were approved by the Ethical Committee for Animal Studies at Zhejiang University.

### In Vivo CS Exposures and Treatments

Mice were exposed to CS in a stainless-steel chamber using a whole-body smoke exposure system (TE-10, Teague Enterprises, Woodland, CA, USA) for approximately 2 h per day (50 cigarettes), 5 days per week. Total particulate matter concentrations in the exposure chamber were between 150-180 μg/m^3^. Serum cotinine levels measured immediately after CS exposure were around 20 ng/ml. Control groups were exposed to filtered room air.

Mouse elastin peptide was suspended in sterile saline at 2 mg/ml. A total of 100 μg elastin peptide in 50 μl saline was administered intratracheally on each indicated experimental day.

For the CS+Eln experimental outline: Mice were exposed to CS or room air for 2 weeks and were hosted at room air for another 2 weeks. Mice were then challenged with 100 μg elastin peptide or normal saline (NS) intratracheally for 3 times at day 29, 30, and 31, and were sacrificed 48 h after the last elastin challenge.

3-MA at 4 mg/ml in NS was delivered intratracheally using a micro-syringe on each indicated experimental day, NS serves as control. Doxycycline at 2 mg/ml was deliver through drinking water during CS sensitization from d1 to d14, and clean drinking water serves as control.

### Transmission Electron Microscopy

For transmission electron microscopy analysis, mouse bronchus or distal lung tissues were fixed in 2.5% glutaraldehyde in PBS for 24 h after experimental manipulations. These samples were washed in PBS, post-fixed in 1% osmium tetroxide, and stained with 4% uranyl acetate. The samples were embedded in embedding medium after being dehydrated. Ultrathin sections were stained with uranyl acetate and lead citrate. Images were taken using a TECNA1 10 transmission electron microscope (FEI, Hillsboro, OR, USA) at 80 kV. To quantify the alteration of the number of the autophagic vacuoles (AVs), the area of the cell cytoplasm was measured by using Image-Pro Plus 6.0 (Media Cybernetics, Inc., Rockville, MD, USA). The data were represented as AVs per 100 μm^2^.

### Bronchial Alveolar Lavage Fluid Collection and Analysis

Forty-eight hours after the last exposure to elastin, mice were sacrificed using an overdose of 2% pentobarbital sodium and tracheotomy was performed. Each mouse was lavaged with 0.4 ml PBS by injecting into the lungs and drawing to collect cells for 3 times. The total number of bronchial alveolar lavage fluid (BALF) cells was counted, then the remaining BALF was centrifuged (3000 g for 10 min at 4°C). The supernatant was retained for further analysis, while the cell pellet was resuspended in PBS moderately and centrifuged on glass slides. Then cells on glass slides were stained with Wright–Giemsa stain, and differential counts were assessed by counting 200 total cells.

### Histological Analyses

The left lobes of lungs were fixed in 4% paraformaldehyde at 4°C for 24 h. After paraffin embedding, the tissue sections were prepared (3 μm). Lung sections were stained with hematoxylin/eosin (H&E) or Periodic acid–Schiff (PAS) following standard protocol (25), Inflammation was assessed according to published guidelines (26), and PAS stained goblet cells in airway epithelium were scored as described previously (25, 26).

### Western Blot Assay

The lysates of cigarette smoke CSE-treated THP-1 cells and lung tissue were prepared with RIPA buffer (P0013B, Beyotime, Shanghai, China) containing protease (04-693-116-001, Roche Diagnostics GmbH, Mannheim, Germany) and phosphatase inhibitors (04-906-837-001, Roche Diagnostics GmbH, Mannheim, Germany). The supernatants of lysates were run on gels and incubated with relevant antibodies using standard methods. ACTB was used as a loading control. Quantification was performed by densitometry and analyzed using Image Studio Lite (LI-COR Biosciences, Lincoln, NE, USA).

### RNA Isolation and Quantitative Real-Time PCR Analysis

RNA from THP-1 cells, BMDMs and lung homogenates were isolated using RNAiso Plus (9109, Takara Bio, Kusatsu, Shiga, Japan). After isolation, the quality of the RNA was assessed with the NanoOne Spectrophotometer (Thermo Fisher Scientific, Waltham, MA, USA) according to the manufacturer’s instructions. The 260/280 absorbance ratios of 1.8–2.0 indicated a pure RNA sample. Reverse transcription was performed with Reverse Transcription Reagents (DRR037A, Takara Bio, Kusatsu, Shiga, Japan). The expressions of mouse *Cxcl1*, *Cxcl2*, *Il6*, *Ifng*, *Mmp12* and human *MMP12* were measured by quantitative real-time PCR using SYBR Green Master Mix (DRR041A, Takara Bio, Kusatsu, Shiga, Japan) on a StepOne real-time PCR system (Applied Biosystems, Foster City, CA, USA). All protocols were performed according to the manufacturer’s instructions. QPCR primers (mouse *Cxcl1* Forward 5’-3’: CTG GGA TTC ACC TCA AGA ACA TC, Reverse 5’-3’: CAG GGT CAA GGC AAG CCT C; mouse *Cxcl2* forward 5’-3’: TGT CCC TCA ACG GAA GAA CC, Reverse 5’-3’: CTC AGA CAG CGA GGC ACA TC; mouse *Il6* forward 5’-3’: CTG CAA GAG ACT TCC ATC CAG, Reverse 5’-3’: AGT GGT ATA GAC AGG TCT GTT GG; mouse *Ifng* forward 5’-3’: GCC ACG GCA CAG TCA TTG A, Reverse 5’-3’: TGC TGA TGG CCT GAT TGT CTT; mouse *Muc5ac* forward 5’-3’: CTG TGA CAT TAT CCC ATA AGC CC, Reverse 5’-3’: AAG GGG TAT AGC TGG CCT GA; mouse *Mmp12* forward 5’-3’: CAT GAA GCG TGA GGA TGT AGA C, Reverse 5’-3’: TGG GCT AGT GTA CCA CCT TTG; human *MMP12* forward 5’-3’: CAT GAA CCG TGA GGA TGT TGA, Reverse 5’-3’: GCA TGG GCT AGG ATT CCA CC) were synthesized by Sangon Biotech, Shanghai, China.

### ELISA

The concentration of CXCL1, CXCL2, IL6 and IFNg in BALF supernatants were measured by ELISA kits following the manufacturer’s protocol. ELISA kits for mouse CXCL1 (MKC00B), mouse CXCL2 (MM200), mouse IL6 (M6000B) and mouse IFNg (MIF00) were purchased from R&D systems (Minneapolis, MN, USA).

### Statistics

One-way analysis of variance (ANOVA) was used to analyze the statistical differences among three or more groups, with *P* values indicated in the related graphs. Differences between 2 groups were identified using the Student’s *t*-test. All data are expressed as mean ± s.e.m. The analyses and graphs were performed using GraphPad Prism 8.0 software (GraphPad Software Inc., San Diego, CA, USA). A value of *P* less than 0.05 was considered statistically significant.

## Results

### CS Exposure Following Elastin Challenge Elicits Autophagy in Mouse Lung Tissues

**Figure 1:**
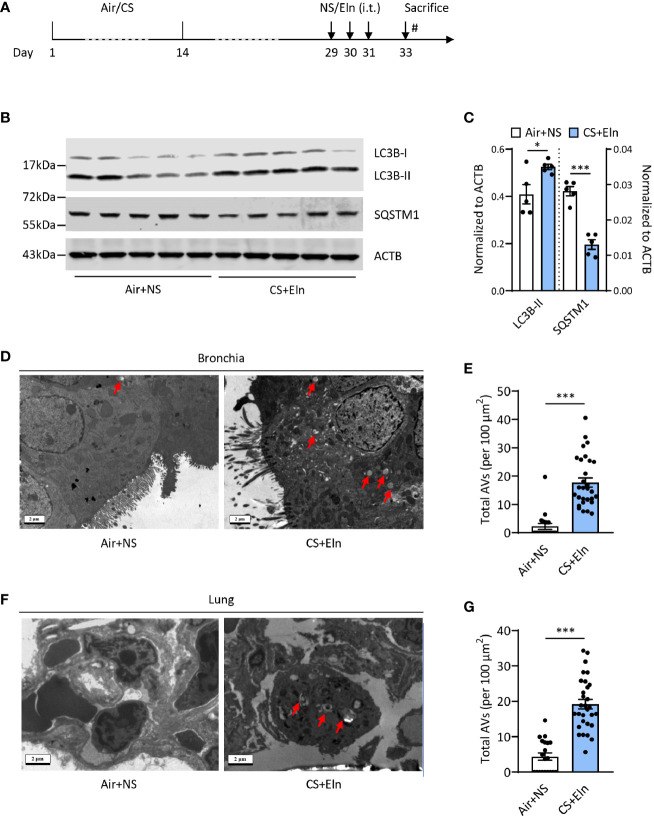
CS exposure following elastin challenge elicits autophagy in mouse lung tissues. **(A)** Experimental outline. Mice were exposed to CS or room air for 2 weeks and were hosted at room air for another 2 weeks. Mice were then challenged with elastin peptide (Eln, 100 μg) or normal saline (NS) intratracheally (i.t.) for 3 times at day 29, 30, and 31, and were sacrificed 48 h after the last elastin challenge. **(B)** Representative immunoblots and **(C)** semi-quantification of LC3B and SQSTM1 in lung tissues of CS+Eln mouse models, ACTB serves as a loading control. **(D, F)** TEM images (n = 20 images for Air+NS group and n = 30 images for CS+Eln group) and **(E, G)** semi-quantified level of autophagic vacuoles in isolated mouse bronchus and lung tissue respectively. Red arrowheads denoted autophagic vacuoles. Scale bar = 2 μm. Data are presented as mean ± s.e.m. Differences between two groups were identified using the Student t-test *p < 0.05, ***p < 0.001.

To study the possible role of autophagy in the new COPD model induced by CS sensitization and elastin challenge (hereafter referred as CS+Eln model, **Figure 1A**), we first examined the expression of autophagy in lung tissues of this model. As expected, microtubule-associated protein 1 light chain 3B (LC3B) was increased, along with decreased Sequestosome 1 (SQSTM1), in lung tissues of the CS+Eln model (**Figures 1B, C**). The increased autophagy in lung tissues was further confirmed by transmission electron microscopy. As shown in **Figures 1D–G**, autophagic vacuoles (AVs) were clearly increased in bronchial epithelium as well as in lung parenchyma cells.

### Impairment of Autophagic Proteins Attenuates the Bronchitis-Like Phenotypes in the CS+Eln Model

**Figure 2:**
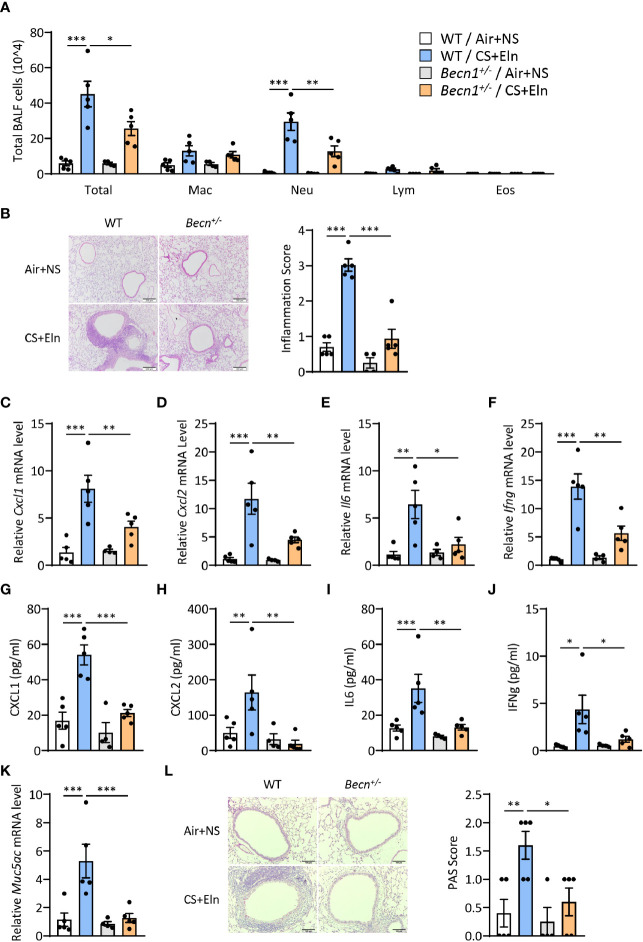
Impairment of *Becn^+/-^* attenuates the bronchitis-like phenotypes in the CS+Eln model. Experimental outline was the same as **Figure 1A**, **(A)** Inflammatory cell counts in the BALF. **(B)** Representative images and semi-quantified scoring of H&E staining of mouse lung sections. Scale bar = 200 μm. **(C–F)** The *Cxcl1*, *Cxcl2*, *Il6* and *Ifng* mRNA transcripts in lungs. **(G–J)** The protein concentrations of CXCL1, CXCL2, IL6 and IFNg in BALF. **(K)** Expression of *Muc5ac* mRNA transcripts in mouse lungs. **(L)** Representative images and the semi-quantified scorings of PAS staining in mouse lung sections. Scale bar = 100 μm. Labeling for all the columns throughout **Figure 2** was shown in **(A)**. Mac, macrophages; Neu, neutrophils; Lym, lymphocytes; Eos, eosinophils. Throughout, data are representative of 4-5 mice and were replicated in at least 3 independent experiments. Data are presented as mean ± s.e.m. *p < 0.05, **p < 0.01, ***p < 0.001 by one-way ANOVA.

We next aimed to explore the function of autophagy in the airway inflammation in the CS+Eln model. *Becn1^+/-^* and *Lc3b^-/-^* mice, which exhibit impaired autophagy in lungs and other organs (10, 11, 16), were used for the *in vivo* study. In line with the findings from traditional chronic CS model, airway inflammation induced by CS+Eln was significantly decreased in these autophagy impaired mice relative to their littermate controls (**Figures 2A, B** and **Supplemental Figure 1A**). Inflammatory cytokines such as CXCL1, CXCL2, IL6, and IFNg were also notably reduced in *Becn1^+/-^* mice (**Figures 2C–J**, **Supplemental Figures 1B–E**). Mucus hyperproduction, as evidenced by expression of *Muc5ac* mRNA in lung tissues and the positive PAS staining in bronchial epithelium, was also markedly attenuated in *Becn1^+/-^* mice (**Figures 2K, L**).

### Inhibition of Autophagy Either During Elastin Challenge pr During CS Sensitization Alleviates the Neutrophilic Airway Inflammation *In Vivo*

**Figure 3:**
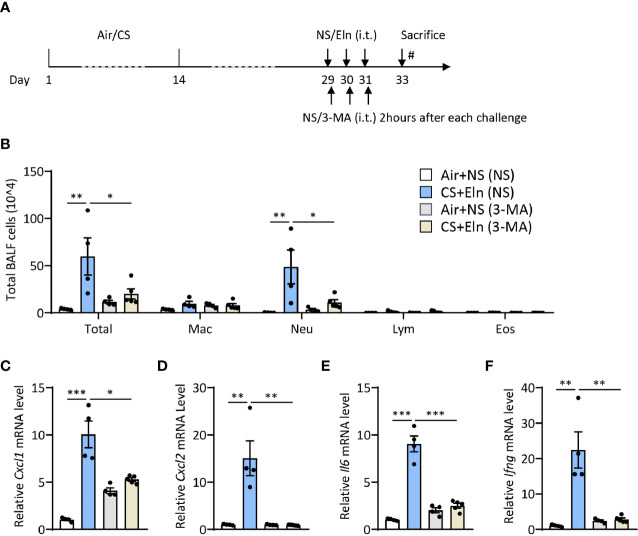
Inhibition of autophagy during elastin challenge alleviates the neutrophilic airway inflammation *in vivo*. **(A)** Experimental outline and protocol for 3-MA treatment, 3-MA (4 mg/ml, 50 μl) was administrated intratracheally 2 hrs after each elastin peptide (2 mg/ml, 50 μl) challenge. **(B)** Inflammatory cell counts in the BALF. **(C–F)** The *Cxcl1*, *Cxcl2*, *Il6* and *Ifng* mRNA transcripts in lungs. Labeling for all the columns throughout **Figure 3** was shown in **(B)**. Mac, macrophages; Neu, neutrophils; Lym, lymphocytes; Eos, eosinophils. Throughout, data are representative of 4-5 mice and were replicated in at least 3 independent experiments. Data are presented as mean ± s.e.m. *p < 0.05, **p < 0.01, ***p < 0.001 by one-way ANOVA.

**Figure 4:**
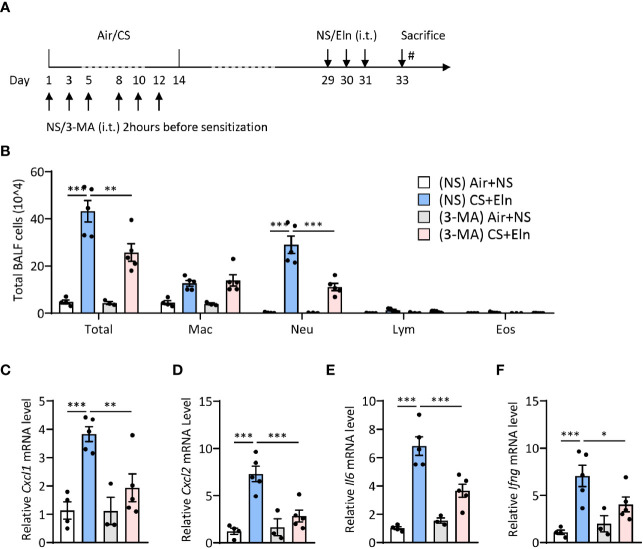
Inhibition of autophagy during CS sensitization alleviates the neutrophilic airway inflammation *in vivo.* **(A)** experimental outline and protocol for 3-MA treatment, 3-MA (4 mg/ml, 50 μl) was administrated intratracheally 2 hrs before each indicated CS exposure day. **(B)** Inflammatory cell counts in the BALF. **(C–F)** The *Cxcl1*, *Cxcl2*, *Il6* and *Ifng* mRNA transcripts in lungs. Labeling for all the columns throughout **Figure 4** was shown in **(B)**. Mac, macrophages; Neu, neutrophils; Lym, lymphocytes; Eos, eosinophils. Throughout, data are representative of 3-5 mice and were replicated in at least 3 independent experiments. Data are presented as mean ± s.e.m. *p < 0.05, **p < 0.01, ***p < 0.001 by one-way ANOVA.

Since the overall protective effect in *Becn1^+/-^* and *Lc3b^-/-^* mice in the CS+Eln model could not specialize the exact phase where autophagy exerted its effect, we then utilized 3-MA to inhibit autophagy at different stages. As **Figure 1** has shown the increased autophagy in airway epithelium and lung parenchyma, we first treated the mice with 3-MA at 2 h after each elastin provocation (**Figure 3A**). As expected, the autophagy inhibitor markedly diminished the neutrophilic airway inflammation in BALF (**Figure 3B**) as well as the inflammatory cytokines in lung tissues (**Figures 3C–F**). Next, we treated the mice with 3-MA during sensitization at 2h before each CS exposure (**Figure 4A**). Intriguingly, inhibition of autophagy at this stage also notably ameliorated the subsequent elastin-induced airway inflammation (**Figures 4B–F**).

### Induction of MMP12 by CS During Sensitization Phase Is Critical for the Ensuing Airway Inflammation Induced by Elastin

**Figure 5:**
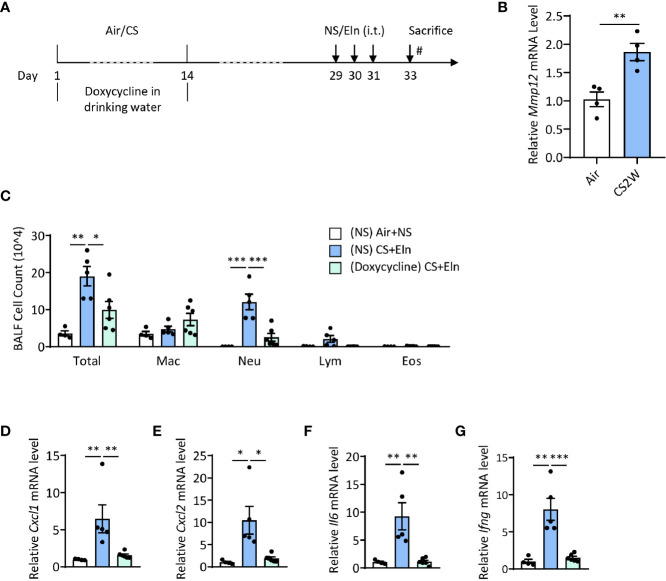
Induction of MMP12 by CS during sensitization phase is critical for the ensuing airway inflammation induced by elastin. **(A)** experimental outline and protocol for doxycycline treatment, mice were fed with doxycycline (2 mg/ml) in drinking water during CS sensitization phase. **(B)** Expression of *Mmp12* mRNA transcripts in mouse BALF cells. **(C)** Inflammatory cell counts in the BALF. **(D–G)** The *Cxcl1*, *Cxcl2*, *Il6* and *Ifng* mRNA transcripts in lungs. Labeling for all the columns throughout **Figure 5** was shown in **(C)**. Mac: macrophages; Neu: neutrophils; Lym: lymphocytes; Eos: eosinophils. Dox: doxycycline. Throughout, data are representative of 4-6 mice and were replicated in at least 3 independent experiments. Data are presented as mean ± s.e.m. Difference between two groups was identified using the Student t-test **(B)** and multiple groups using one-way ANOVA **(C–G)**. *p < 0.05, **p < 0.01, ***p < 0.001.

We have utilized *Mmp12^-/-^* mice to demonstrate the pivotal role of this metalloproteinase in driving the autoimmunity in the CS+Eln model (23). Again, this overall genetic knockout of MMP12 could not specialize whether MMP12 exerted its function in the sensitization phase. We then treated the mice with doxycycline, a pan metalloproteinase inhibitor, during CS exposure (**Figure 5A**). It was not surprising that CS rapidly induced MMP12 expression in mouse alveolar macrophages (**Figure 5B**). In line with the findings from *Mmp12^-/-^* mice (23), inhibition of metalloproteinase activity in the sensitization phase also notably reduced the neutrophilic airway inflammation (**Figures 5C–G**).

### Autophagy Promotes CS-Induced MMP12 Expression in Macrophages

**Figure 6:**
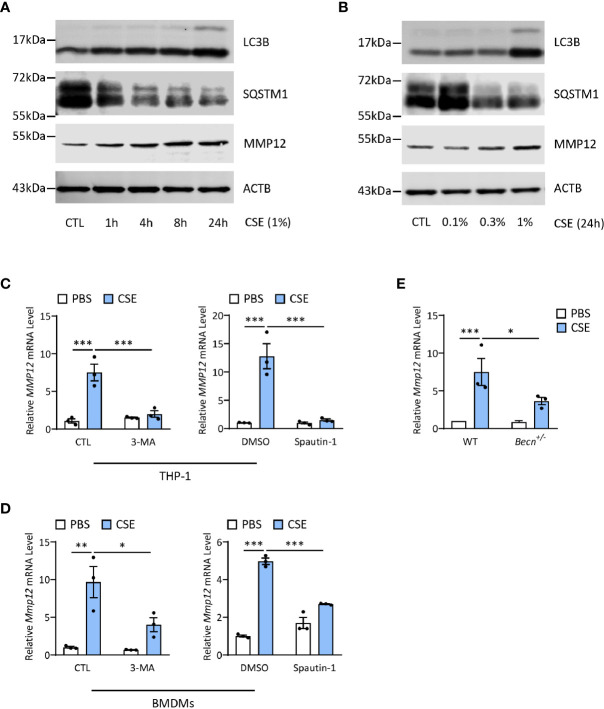
Autophagy promotes CS-induced MMP12 expression in macrophages. **(A)** Representative immunoblots of time-dependent expression of LC3B and MMP12 in THP-1 cells exposed to 1% CSE. **(B)** Representative immunoblots dose-dependent expression of LC3B and MMP12 in THP-1 cells exposed to CSE for 24 h. **(C)** Expression of *MMP12* mRNA transcripts in THP-1 cells treated with 3-MA (4 mM) or Spautin-1 (10 μM) along with 1% CSE for 24 h. **(D)** Expression of *Mmp12* mRNA transcripts in BMDMs treated with 3-MA (4 mM) or Spautin-1 (10 μM) along with 1% CSE for 24 h. **(E)** Expression of *Mmp12* mRNA transcripts in BMDMs from *Becn^+/-^* mice. Throughout, data were replicated in at least 3 independent experiments. Data are presented as mean ± s.e.m. *p < 0.05, **p < 0.01, ***p < 0.001 by one-way ANOVA.

Last, we asked whether autophagy played any functions in regulation of MMP12 expression during CS sensitization. Interestingly, CSE could increase both LC3B and MMP12 expression in a time- and dose-dependent manner in THP-1 cells, a human monocyte-derived macrophage cell line (**Figures 6A, B**), without affecting the cell viability (**Supplementary Figures 2A, B**). Inhibition of autophagy by 3-MA or spautin-1, the latter of which promotes the degradation of Vps34 PI3 kinase complexes by inhibiting USP10 and USP13 (27), remarkably shut down the *MMP12* transcripts induced by CSE in THP1 or BMDMs (**Figures 6C, D**). Similar results could be found in autophagy-impaired *Becn^+/-^* BMDMs (**Figure 6E**). Again, without affecting the cell viability (**Supplementary Figures 2C–E**).

## Discussion

The major findings of this study can be summarized as follows: 1) Autophagic markers were increased in airway epithelium and lung tissues, and impairment of autophagy reduced the neutrophilic airway inflammation and mucus hyperproduction in the CS+Eln model; 2) treatment of an autophagic inhibitor 3-MA either during CS-initiated sensitization or elastin provocation significantly inhibited the bronchitis-like phenotypes in mice; 3) autophagy mediated MMP12 activation in macrophages during the sensitization played a pivotal role in this novel mouse model of COPD.

Pulmonary epithelial cells, along with alveolar macrophages, comprise the first line of the host innate immune response and play a crucial role during early CS exposure. Autophagy is a revolutionary conserved ubiquitous process by which cytoplasmic materials, including soluble macromolecules and organelles, are delivered to lysosomes for degradation when facing environmental and/or developmental changes, with connections to human physiology and disease (8, 9). Emerging evidence suggests that autophagy plays pivotal but controversial roles in pulmonary epithelial injury upon CS exposure. Even though autophagy impairment in lung parenchyma may contribute to cell senescence, lung aging, and emphysema (19–22, 28, 29), it is believed that autophagy appears to worsen the epithelial injury induced by CS. We initially had reported that CS-induced autophagy could mediate the cell apoptosis in airway epithelial cells (10, 11), and further explored that caveolin-1 was tightly involved in regulating autophagy homeostasis by competitively interacting with the ATG12-ATG5 system to suppress the formation and function of the latter in lung epithelial cells (30). Later, we observed that the starvation or Torin 1 treatment was capable of inducing MUC5AC expression, and inhibition of autophagy markedly attenuated cigarette smoke extract (CSE)-induced mucus production (12). In line with these observations, our current findings indicated that genetic impairment of autophagic proteins Beclin-1 or LC3B, or chemical inhibition of autophagy either during CS-initiated sensitization or during elastin provocation significantly inhibited the bronchitis-like phenotypes in mice, reassuring the deleterious effect of autophagy in CS-induced epithelial injury.

It is noteworthy that we utilized our recently developed novel CS+Eln mouse model to investigate the roles of autophagy in different stages of the development of bronchitis-like airway inflammation throughout the current study. Surely this model is not good for the direct study of epithelial injury since it is conceived on the basis of elastin-mediated autoimmunity in COPD pathogenesis. However, we have demonstrated that no inflammation was observed in mice exposed to CS and challenged with vehicle (normal saline) or provocation by elastin alone without CS sensitization hardly reproduce airway inflammation (23), suggesting that inflammation was related to the combination of CS exposure and elastin administration, rather than inflammatory processes driven by elastin instillation alone or residual inflammation following two weeks of smoke exposure. Therefore, this CS+Eln model is ideal for COPD inflammation research since it is less time-consuming with more reliable and consistent data than the traditional CS exposure. One thing worth mentioning is that sex differences in the risk of developing COPD have been well described. In fact, females are more likely to develop COPD upon tobacco exposure (31–33). We have tested the response of female mice in our current CS+Elastin COPD model, and there was no significant difference observed between the genders. However, we used male mice which are less influenced by sex hormones in our animal research.

Alveolar macrophages play pivotal roles in maintenance of tissue homeostasis, host defense, promotion of immunological tolerance, and anti-inflammatory response. Nowadays, more attention is focused on the functions of autophagy in macrophages. Signaling of Toll-like receptor (34) and TIM-4 glycoprotein (35) link pathways of autophagy and phagocytosis, and enhancement of autophagy promotes clearance of the apoptotic cells by macrophages (36, 37). Therefore, it is not surprising that in most of the cases, LC3-associated phagocytosis protects against inflammation, and other pathophysiological condition including liver fibrosis and neurodegeneration (38–40). Monick and colleagues have demonstrated that CS impaired autophagy in alveolar macrophages of COPD patients, and this autophagy impairment resulted in decreased protein aggregate clearance, dysfunctional mitochondria, and defective delivery of bacteria to lysosomes (22). However, the present study suggested that autophagy played a deleterious role in macrophages in the context of COPD pathogenesis. Inhibition of autophagy during CS-initiated sensitization attenuated the subsequent elastin-induced airway inflammation *in vivo*, and this effect was most likely due to autophagy mediated MMP12 production as demonstrated *in vitro*. It is plausible to assume that the controversial observations might be due to the different exposure time of macrophages to these outside irritants. In the current study we detected the autophagy pathway in a relative short exposure period (two weeks of CS exposure plus two weeks in room air), while the human alveolar macrophages obtained from COPD patients were likely chronically exposed to CS. On the other hand, although we have not tested the exact phagocytosis property of lung macrophages in our smoke exposure strategy, it is tempting to speculate that functional properties of lung macrophages including phagocytosis may be impaired due to exposure to CS (41, 42). Currently it is not clear on the exact mechanism how enhanced autophagic flux in macrophage upon CS exposure resulted in seemingly impaired phagocytosis.

It is widely acknowledged that MMP12 is essential for the development of emphysema in mice exposed to CS due to its massive capacity to degrade extracellular matrix (43–45). However, our recent study applied *Mmp12^-/-^* mice to demonstrate the pivotal role of this metalloproteinase in driving the autoimmunity in the CS+Eln model, indicating that aside from emphysema, MMP12 may also contribute to the development of airway inflammation (23). Here, we intended to further specialize whether MMP12 exerted its function in the sensitization phase and discovered that inhibition of metalloproteinase activity during CS exposure notably alleviated the ensuing elastin-induced airway inflammation. In addition, *in vitro* studies on THP-1 and BMDMs showed that chemical inhibition of autophagy or genetic impairment of autophagic protein Beclin1 could prominently dampen Mmp12 transcription. To the best of our knowledge, this is the first time showing that autophagy is linked to the promotion of MMP12 expression in macrophage. The detailed molecular mechanisms how autophagy regulates MMP12 expression remains unclear. We have demonstrated that autophagy could regulate airway inflammation through nuclear factor kappa B (NFKB) activation (13, 16), and it has been shown that MMP12 could be a downstream targeted gene of NFKB signaling (46). Thus, autophagy is likely to regulate MMP12 expression through the NFKB pathway in macrophages, which needs to be demonstrated in future study.

Our new animal model represents two specific stages (CS sensitization and elastin provocation) for development of the COPD-like airway inflammation; however, an overall genetic knockout model could not differentiate the eventual functions of the protein in this model. Thus, our current study used chemical compounds such as 3-MA and doxycycline at different stages of our new model. Although chemical inhibitors might exert non-specific effects, they indeed represent potentials for clinical drug development. It is worth mentioning that the effects of those inhibitors were in line with the corresponding genetic-engineered mice, which validated their specific functions *in vivo*.

In conclusion, our findings demonstrate that autophagy promoted the bronchitis-like airway inflammation, likely through facilitation of both early MMP12 induction in macrophages and late airway epithelial injury. Moreover, this study reemphasizes that inhibition of autophagy as a novel therapeutic strategy for CS-induced COPD.

## Data Availability Statement

The raw data supporting the conclusions of this article will be made available by the authors, without undue reservation.

## Ethics Statement

The animal study was reviewed and approved by the Ethical Committee for Animal Studies at Zhejiang University.

## Author Contributions

H-QH, ZhoL, H-HS, and Z-HC conceived and designed the experiments. H-QH, NL, D-YL, DJ, ZheL, X-CX, H-PC, MZ, L-LD, and ZhoL performed the experiments and analyzed the data. Z-HC wrote the manuscript with assistance of H-QH and ZhoL. H-QH, NL, WL, SY, H-HS, ZhoL, and Z-HC discussed and interpreted the results. D-YL, ZhoL, and Z-HC revised the manuscript. All authors contributed to the article and approved the submitted version.

## Funding

This work is supported by the National Key R&D Program of China (2016YFA0501802 to Z-HC and 2017YFC1310604 to H-QH), and the Key (81930003 to H-HS) and General (81670031 and 31970826 to Z-HC.) Projects from the National Natural Science Foundation of China.

## Conflict of Interest

The authors declare that the research was conducted in the absence of any commercial or financial relationships that could be construed as a potential conflict of interest.
