# Corrigendum: Autophagy Promotes Cigarette Smoke-Initiated and Elastin-Driven Bronchitis-Like Airway Inflammation in Mice

**DOI:** 10.3389/fimmu.2021.772939

**Published:** 2021-09-27

**Authors:** Hua-Qiong Huang, Na Li, Dan-Yang Li, Du Jing, Zheng-Yuan Liu, Xu-Chen Xu, Hai-Pin Chen, Ling-Ling Dong, Min Zhang, Song-Min Ying, Wen Li, Hua-Hao Shen, Zhou-Yang Li, Zhi-Hua Chen

**Affiliations:** ^1^ Key Laboratory of Respiratory Disease of Zhejiang Province, Department of Respiratory and Critical Care Medicine, Second Affiliated Hospital of Zhejiang University School of Medicine, Hangzhou, China; ^2^ State Key Laboratory of Respiratory Disease, Guangzhou Medical University, Guangzhou, China

**Keywords:** autophagy, chronic obstructive pulmonary disease-COPD, MMP12, inflammation, elastin

There is an error in the **Funding** statement. The correct number for National Key R&D Program of China to Z-HC is “2016YFA0501802”.

The authors apologize for this error and state that this does not change the scientific conclusions of the article in any way. The original article has been updated.

## Publisher’s Note

All claims expressed in this article are solely those of the authors and do not necessarily represent those of their affiliated organizations, or those of the publisher, the editors and the reviewers. Any product that may be evaluated in this article, or claim that may be made by its manufacturer, is not guaranteed or endorsed by the publisher.

