# Supplementary figures and images for: Autophagy Promotes Cigarette Smoke-Initiated and Elastin-Driven Bronchitis-Like Airway Inflammation in Mice
Source: Front Immunol. 2021 Mar 22;12:594330. doi: 10.3389/fimmu.2021.594330 (PMC8019710; doi:10.3389/fimmu.2021.594330)

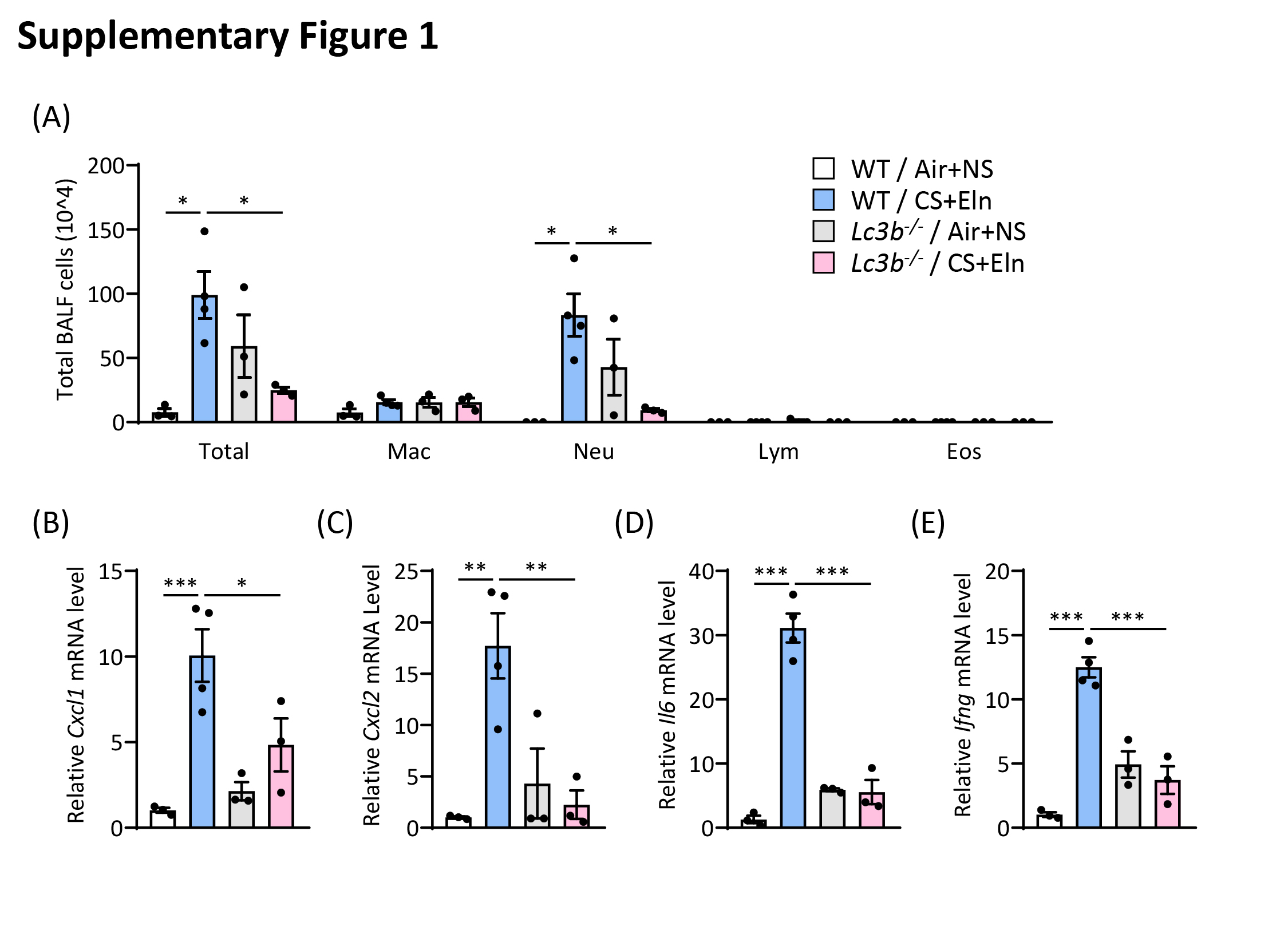

Supplement: Supplementary file 1 [file Image_1.jpeg]

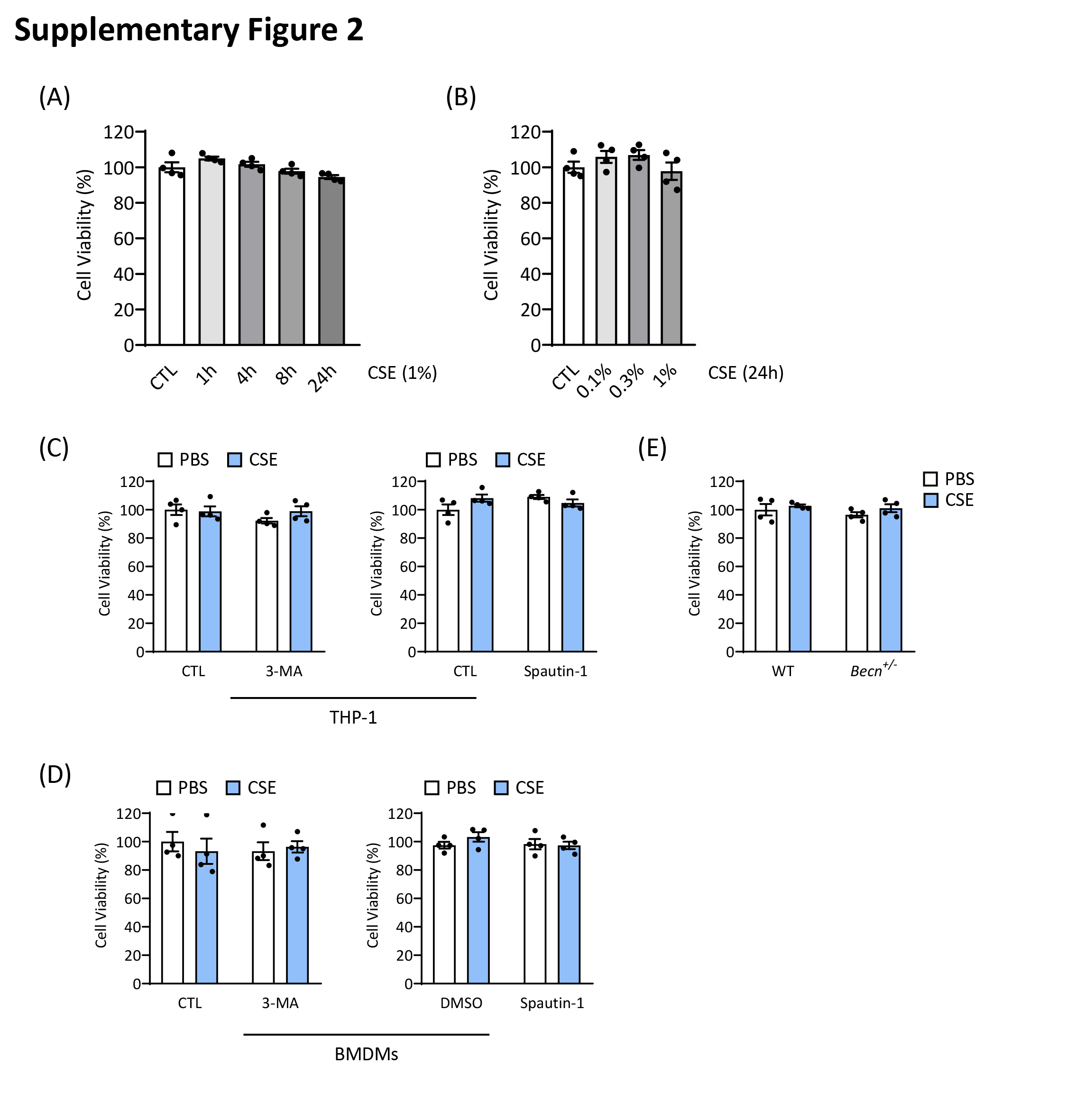

Supplement: Supplementary file 2 [file Image_2.jpeg]
