# Supplementary material for: Autophagy Promotes Cigarette Smoke-Initiated and Elastin-Driven Bronchitis-Like Airway Inflammation in Mice
Source: Front Immunol. 2021 Mar 22;12:594330. doi: 10.3389/fimmu.2021.594330 (PMC8019710; doi:10.3389/fimmu.2021.594330)
Supplement: Supplementary file 3 [file Table_1.docx]

**Supplementary Figure legend**

**Supplementary Figure 1. Impairment of *Lc3b^+/-^* attenuates the bronchitis-like phenotypes in the CS+Eln model. (A)** Inflammatory cell counts in the BALF. **(B to E)** The *Cxcl1*, *Cxcl2*, *Il6* and *Ifng* mRNA transcripts in lungs. Labelling for all the columns throughout Supplementary fig. 1 was shown in Supplementary fig. 1A. Mac: macrophages; Neu: neutrophils; Lym: lymphocytes; Eos: eosinophils. Throughout, data are representative of 3-4 mice and were replicated in at least 3 independent experiments. Data are presented as mean ± s.e.m. * *p* < 0.05, ** *p* < 0.01, *** *p* < 0.001 by one-way ANOVA.

**Supplementary Figure 2. Cell viability of THP-1 cells upon CSE treatment. (A)** Cell viability of THP-1 cells exposed to 1% CSE at indicated time or **(B)** indicated concentration of CSE for 24 h. Cell viability of THP-1 cells **(C)** or BMDMs **(D)** exposed to 1% CSE with or without 3-MA (4 mM, left panels) or Spautin-1 (10 μM, right panels) for 24h. **(E)** Cell viability of WT and *Becn^+/-^* BMDMs treated with 1% CSE for 24h. Data are presented as mean ± s.e.m
